# Mesenchymal stem cells preconditioned with a TLR5 agonist enhanced immunoregulatory effect through M2 macrophage polarization in a murine graft-versus-host disease model

**DOI:** 10.7150/ijms.93121

**Published:** 2024-06-17

**Authors:** Sojin Gil, Keon-Il Im, Nayoun Kim, Junseok Lee, Hyemin Na, Gi-June Min, Seok-Goo Cho

**Affiliations:** 1 Institute for Translational Research and Molecular Imaging, The Catholic University of Korea, Seoul, Republic of Korea.; 2 Department of Biomedicine & Health Sciences, College of Medicine, The Catholic University of Korea, Seoul, Republic of Korea.; 3 Department of Hematology, Seoul St. Mary's Hematology Hospital, College of Medicine, The Catholic University of Korea, Seoul, Republic of Korea.

**Keywords:** Mesenchymal stem cells, Toll-like receptor 5, immunoregulation, M2 macrophage, graft versus host disease.

## Abstract

Graft-versus-host disease (GVHD) is a common complication following hematopoietic stem cell transplantation and can be life-threatening. Mesenchymal stem cells (MSCs), adult stem cells with immunomodulatory properties, have been used as therapeutic agents in a variety of ways and have demonstrated efficacy against acute GVHD (aGVHD); however, variability in MSC pro- and anti-inflammatory properties and the limitation that they only exhibit immunosuppressive effects at high levels of inflammation have prevented their widespread clinical use. The outcomes of GVHD treated with MSCs in the clinic have been variable, and the underlying mechanisms remain unclear. Therefore, the unique biological effects of Toll-like receptor 5 (TLR5) agonists led us to compare and validate the efficacy of MSCs primed with KMRC011, a TLR5 agonist. KMRC011 is a stimulant that induces the secretion of cytokines, which play an important role in immune regulation. In this study, we found that MSCs pretreated with KMRC011 increased the secretion of immunosuppressive cytokines indoleamine 2,3-dioxygenase (IDO) and cyclooxygenase-2 (COX2) and increased the expression of M2 macrophage polarizing cytokines macrophage colony-stimulating factor (M-CSF) and interleukin 10 (IL-10) *in vitro*. We investigated the immunosuppressive effects of TLR5 agonist (KMRC011)-primed MSCs on lymphocytes and their preventive and therapeutic effects on an *in vivo* mouse aGVHD model. *In vitro* experiments showed that KMRC011-primed MSCs had enhanced immunosuppressive effects on lymphocyte proliferation. *In vivo* experiments showed that KMRC011-primed MSCs ameliorated GVHD severity in a mouse model of induced GVHD disease. Finally, macrophages harvested from the spleens of mice treated with KMRC011-primed MSCs showed a significant increase in the anti-inflammatory M2 phenotype. Overall, the results suggest that KMRC011-primed MSCs attenuated GVHD severity in mice by polarizing macrophages to the M2 phenotype and increasing the proportion of anti-inflammatory cells, opening new horizons for GVHD treatment.

## Introduction

Graft-versus-host disease (GVHD) is a serious inflammatory disease caused by an immune-mediated attack of recipient tissue by donor T cells during transplantation. Without intervention before or after allogeneic hematopoietic stem cell transplantation (HSCT), nearly all allogeneic stem cell transplant recipients develop severe graft-versus-host disease. 1 GVHD is a major cause of increased mortality following allogeneic hematopoietic stem cell transplantation; there are many other causes of mortality in patients, including infection and toxicity, which are complications of GVHD. Various cellular therapies have been introduced to address the issue, and several cell-based therapeutic approaches, including mesenchymal stem cells (MSCs), have proven effective in reducing acute GVHD (aGVHD) complications and improving survival following allogeneic hematopoietic stem cell transplantation. 1-3 Although the use of MSCs as a cell therapy has shown promising initial results, long-term overall survival has not been encouraging, as it is no better than that of the control group. 4, 5

MSCs have the most pronounced immunosuppressive capacity in both cell contact-dependent and paracrine environments 6 and express toll-like receptors (TLRs), which are widely distributed on immune cells and serve as key sensors of invading pathogens. 7 MSCs are thought to be immunoprivileged because of their low immunogenicity; they express significantly low levels of major histocompatibility complex (MHC) class I, no MHC class II, and do not induce activation of allogeneic lymphocytes. 8 They have been used to treat various inflammatory or autoimmune diseases because they can contribute to tissue repair. 9, 10 MSCs secrete several soluble factors, including transforming growth factor beta (TGF-β), prostaglandin E2 (PGE2), nitric oxide (NO), and indoleamine 2,3-dioxygenase (IDO) to suppress active T cells 11, 12 and induce M2 macrophages. 13 For MSCs to have an upregulated immunomodulatory effect, they must be primed with interferon-gamma 14 or create an acute inflammatory condition 15; however, in clinical practice, calcineurin inhibitors are commonly used after HSCT 16, 17 and when GVHD occurs, steroids are administered in high doses. 18 Therefore, the immunomodulatory action of MSCs in a low grade inflammatory state does not occur leading to no therapeutic effects. We theorized that inducing anti-inflammatory function even in low grade inflammation would be highly effective in the treatment of other inflammatory diseases, including autoimmune diseases.

KMRC011, a TLR5 agonist, was used to induce anti-inflammatory effects in MSCs, even during low-grade inflammation. TLR5 agonists are known to bind TLR5 and attenuate key pathological processes in bleeding and sepsis caused by hematopoietic and gastrointestinal system injury. 19 Stimulation of TLR5 on MSCs increases signaling by nuclear factor kappa B (NFκB), 20 a transcription factor that plays a key role in cellular and organismal responses to infectious agents as a mediator of innate and adaptive immune responses. NFκB activation induces several factors that contribute to cytoprotection and promotion of tissue regeneration, including apoptosis inhibitors, reactive oxygen species scavengers, and cytokines. 19 NFκB activation via TLR5 agonists does not induce a potentially dangerous inflammatory response, but rather protects against it. 21, 22 Immediate TLR5-dependent effects include production of hematopoietic cytokines such as granulocyte colony stimulating factor (G-CSF) and interleukin (IL)-6, 23 anti-infective factors, 24 and neutrophil mobilization. In addition, stimulation of TLR5 suppresses radiation-induced sterile inflammation associated with secondary tissue damage through the stimulation MSCs, which express the anti-inflammatory cytokines IL-10 and IL-1βa 22 and have anti-inflammatory properties. 21

We found that pretreatment of MSCs with TLR5 agonists prevented their transformation into pro-inflammatory types, a problem in MSC cell therapy, and exerted immunosuppressive effects even in low grade inflammation caused by steroid use. These agonists synergistically suppressed GVHD disease by increasing the expression of immunosuppressive factors by the NFκB signaling pathway and inducing M2 macrophage-associated polarization and migratory cytokine secretion, an anti-inflammatory phenotype.

## Materials and Methods

### Mice

Bagg albino (BALB/c (H-2d) mice (8-10 weeks old) and C57 black (BL)/6 (H-2b) mice were purchased from OrientBio (Sungnam, Korea). The mice were maintained under specific pathogen-free conditions in an animal facility with controlled humidity (55±5%), light (12/12 h light/dark), and temperature (22±1°C). The air in the facility was passed through a high efficiency particulate air filter system designed to exclude bacteria and viruses. Animals were fed mouse chow and tap water ad libitum. The animal care and euthanasia protocols used in this study were approved by the Animal Care and Use Committees at Korea University and the Catholic University of Korea, College of Medicine (Permit number: 2023-0220-01).

### Isolation and culture of MSCs

Bone marrow was isolated from the femur and tibia of C57BL/6 animals and cultured in complete Dulbecco's modified Eagle's medium (Gibco, Carlsbad, CA, USA) containing 10% fetal bovine serum (FBS) (Gibco), 2 mM L-glutamine (Gibco), and 1% antibiotics (penicillin [10 U/mL]-streptomycin [10 g/mL]). We used 10% heat-inactivated FBS with endotoxin levels of less than 5 EU/mL and hemoglobin levels of less than 10 mg/dL. The cellular immunophenotype was consistently positive for spinocerebellar ataxia type 1 (SCA1), cluster of differentiation (CD)44, and CD29, but negative for c- tyrosine-protein kinase (Kit), CD11b, and CD45, even after more than 15 passages using the antibodies described below, which is consistent with previous reports. 25 Cells used in these experiments were present between passages 15-20.

### TLR5 agonist

The TLR5 agonist used in this experiment was KMRC011, which was provided by Connext (Daegu, South Korea) as a lyophilized powder at a total dose of 150 μg/vial. KMRC011 was prepared according to the manufacturer's instructions, and a 150 μg/mL solution was prepared by adding 1 mL of distilled water to the vial. All drugs were dissolved immediately before use, and the remaining volume was discarded. MSCs were treated with TLR5 agonist at 100 ng/mL for 24 h before use and co-stimulated when seeding MSCs at 2.8x10^4^/cm^2^. For the *in vivo* experiments, MSCs pretreated with TLR5 agonist were administered to mice twice, on the day of bone marrow transplantation (BMT) and 4 d later, at 1x10^6^ per dose.

### Flow cytometric analysis

Cells were collected in flow tubes and centrifuged at 1,800 rpm for 3 min to obtain a cell precipitate. Cells were washed twice with flow cytometry staining (FACS) buffer. Each sample was diluted according to the manufacturer's protocol, resuspended, and incubated in the dark at 4°C for 30 min. Intracellular staining was performed using an intracellular staining kit (eBioscience) according to the manufacturer's protocol. Flow cytometry was performed on a LSRFortessa flow cytometer (Becton Dickinson) and analyzed with Flowjo software v10.7.1 (Tristar, Ashland, OR). Mononuclear cells were immunostained with various combinations of the following fluorescence-conjugated antibodies: Ly-6A/E (Sca-1)-phycoerythrin (PE), CD285 (TLR5)-PE, CD29-fluorescein isothiocyanate (FITC), CD117(c-Kit)-FITC, IL-10-Pacific Blue, CD206-PE, F4/80-Alexa Fluor 700 (BioLegend), CD44-PE, mouse IDO (mIDO)- eFluor™ 660 (eBioscience, San Diego, CA, USA), CD11b-FITC, CD45-FITC, IL-4-PE, mouse transforming growth factor β1 (mTGF-β1)-PE, CD86-adenomatous polyposis coli (APC), CD11b-peridinin-chlorophyll-protein (PerCP)-Cyanine 5.5 (BD Biosciences, San Diego, CA, USA), and mouse cyclooxygenase-2 (mCOX2)-FITC (Bioss). Before intracellular cytokine staining, cells were stimulated in a culture medium containing monensin (GolgiStop, 1 μL/mL; BD PharMingen) in a 5% CO_2_, 37°C incubator for 13 h.

### Western blotting

MSCs were stimulated with IFN-gamma and TNF-alpha, 5 ng/mL each, to create an inflammatory environment or not and were primed with TLR5 agonist for 24 h. The culture medium was discarded and washed twice with cold phosphate-buffered saline (PBS). Cells were lysed by adding radioimmunoprecipitation assay lysate to each well and total cellular protein was obtained by centrifugation. After measuring the total protein concentration with a bicinchoninic acid kit (Thermo Scientific, USA), the proteins were separated by 10% sodium dodecyl sulfate-polyacrylamide gel electrophoresis and electrotransferred onto a polyvinylidene difluoride membrane. The membrane was blocked with 5% skim milk for 1 h at room temperature and then incubated with the target protein primary antibody overnight at 4°C. The membrane was washed with tris-buffered saline and Polysorbate 20 (TBST) (osmotic buffered saline consisting of 0.1% Tween-20 solution) and incubated by horseradish peroxidase-conjugated goat anti-rabbit immunoglobulin G (diluted 1:10,000, USA) for 1 h at room temperature. The membrane was washed three times with TBST and detected using an enhanced chemiluminescence (ECL) detection kit and Hyperfilm-ECL reagent (Amersham Pharmacia Biotech, Piscataway, NJ, USA). Relative protein expression levels were analyzed using Image J. Source antibodies: p-p65 (phospho Ser536) (Cell Signaling Technology, Danvers, MA, USA) and β-actin.

### Real-time quantitative polymerase chain reaction (PCR)

Total ribonucleic acid (RNA) was extracted using the RNeasy Micro Kit (Qiagen, Hilden, Germany) according to the manufacturer's protocol. Total RNA (2 μg) was reverse transcribed at 50°C for 2 min and then at 60°C for 30 min. Quantitative PCR was performed using the iQ™ SYBR® Green Supermix and a real-time PCR machine, CFX96 Touch (Bio-Rad, CA, USA), as specified by the manufacturer. The crossover point was defined as the maximum value of the second derivative of the fluorescence curve. Negative controls included all elements of the reaction mixture excluding template deoxyribonucleic acid. For quantification, relative messenger RNA expression of specific genes was obtained using the ΔΔCt method, with normalization to β-actin.

### MSC/Peripheral Blood Mononuclear Cell (PBMC) co-cultures

MSCs (1.25×10^4^) supplemented with 10% FBS, penicillin (100 U/ml), and streptomycin (100 mg/mL) in Roswell Park Memorial Institute 1640 medium (Gibco) containing 20 mM 4-(2-hydroxyethyl)-1-piperazineethanesulfonic acid (Gibco), 2 mM L-glutamine, 10% heat-inactivated FBS, 100 mM sodium pyruvate, and 1% antibiotics [penicillin (10 U/mL)-streptomycin (10 g/mL)] were used. For TLR5 agonist-priming, MSCs were incubated with KMRC011 100 ng/mL for 24 h prior to co-culture and irradiated at 2,000 rads using a 137Cs source (GammaCell 3000 Elan, Nordion, Ontario, Canada) to reduce proliferation. Human PBMCs were isolated by Lymphoprep (STEMCELL Technologies, Vancouver, Canada) from blood samples of healthy volunteer donors and stimulated with anti-CD3 and anti-CD28. PBMCs (1×10^5^) were added at a ratio of 1:8 to a total of 200 μL wells with or without irradiated MSCs. Cells were incubated at 37°C for 4 d and collected for FACS analysis.

### BMT and aGVHD Induction

All recipient BALB/c H-2d mice were exposed to 800 cGy of radiation at a rate of 70 cGy/min via a Mevatron MXE-2 machine (Siemens, New York, USA). The disease induction group, the GVHD group (n=14), was then intravenously injected with 5×106 bone marrow cells and 5×106 spleen cells from donor mice (C57BL/6, H-2b). Mice in the control group (n = 14) were similarly irradiated, but the donor BM and spleen cells were from BALB/c H-2d mice, and GVHD was not induced. After BMT, survival was monitored daily and the extent of clinical aGVHD was assessed every three days of the week using a scoring system that summed changes in five clinical parameters: weight loss, posture, activity, coat texture, and skin integrity. Animals with a score of 7 or more were considered comatose and euthanized 35 d after transplantation.

### Histology

After mouse death or sacrifice, small intestine and liver samples were fixed in 4% formaldehyde solution and embedded in paraffin. For each organ, 5-µm sections were stained with H&E or IHC for histological examination. One pathologist analyzed the slides in a blinded fashion to assess GVHD intensity. Six parameters were scored for small intestines according to a 0- to 5-point scale adapted from Cooke et al. 26 (surface colonocyte lesions or villous blunting, crypt regeneration, crypt epithelial cell apoptosis, crypt loss, lamina propria inflammation, and mucosal ulceration); seven parameters for the liver according a 0- to 3-point scale (portal inflammation, bile ducts lesions, periportal necrosis, endothelialitis, lobular necro-inflammatory activity, zonal necrosis, and sinusoidal lymphocytosis) 26. Scores for each item were added up to provide a total score for each organ, and scores for each target organ were added up to establish a global histological score for each mouse. IHC staining results were scored according to a semi-quantified scoring system described previously. 27 A score of 1 was given for strong nuclear staining in 1%-10% of the epithelium, 2 for 11%-50%, 3 for 51%-80%, and 4 for 81%-100%.

### Statistics

All data were presented as mean ± standard deviation (SD). GraphPad Prism 9 software (CA) was used for statistical analysis, and two-tailed Student's t test was used for statistical comparisons between the two groups. Differences were considered statistically significant at p < 0.05.

## Results

### Treatment of MSCs with KMRC011 increases NFκB signaling

**Figure 1:**
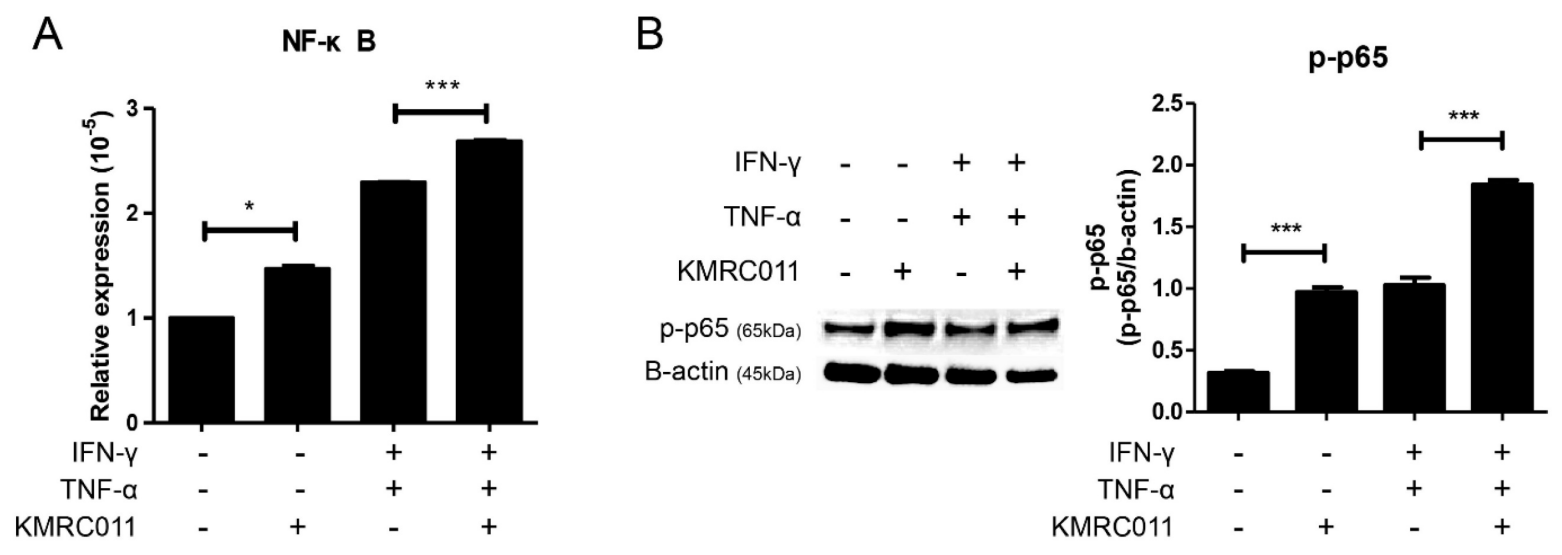
** Increased NF-kB signaling by TLR5 agonist, KMRC011**. Priming MSCs with KMRC011 increases NFκB signal, which was further increased by creating a low-grade inflammation by stimulating IFN-γ and TNF-α at 5 ng/mL each compared to no inflammatory environment.** (A)** Pretreatment of MSCs with TLR5 agonist increased NFκB expression more than MSCs when analyzed by qRT-PCR, and the expression of NFκB significantly increased when treated with TLR5 agonist even when a low-grade inflammation was created. **(B)** NFκB activity was also confirmed when MSCs were treated with TLR5 agonist as analyzed by western blot, and NFκB was significantly activated by TLR5 agonist treatment in a low-grade inflammation. The columns represent the mean values of two independent experiments and the error bars represent standard deviations (SDs). *p < 0.05, **p < 0.01, ***p < 0.001.

Treatment of MSCs with KMRC011 increases signaling through the inhibitor of NFκB kinase pathway. 28 Based on this, we pretreated MSCs with KMRC011 for 24 h, analyzed the increased expression of NFκB, and increased activity signal by quantitative reverse transcription (qRT)-PCR and western blot. The results of qRT-PCR (Figure 1A) showed that the expression of NFκB was increased in the group treated with KMRC011 compared to non-primed MSCs, and when IFN-γ and TNF-α were treated at 5 ng/mL each to create a low grade inflammation, the expression of NFκB was increased compared to when no inflammatory environment was created. Figure 1B (western blot results) shows that p-p65 was identified to confirm the activity of b-actin and NFκB, and it can be seen that the phosphorylation level of p65 was increased in KMRC011-primed MSCs compared to that in non-primed MSCs, as well as in KMRC011-primed MSCs when a low grade inflammation was created. pp65 was identified because TLRs activated the canonical NFκB pathway. Based on the data, TLR5 agonists enhance NFκB signaling.

### KMRC011-MSCs affects cytokine release and expression in MSCs by increasing NFκB signal

**Figure 2:**
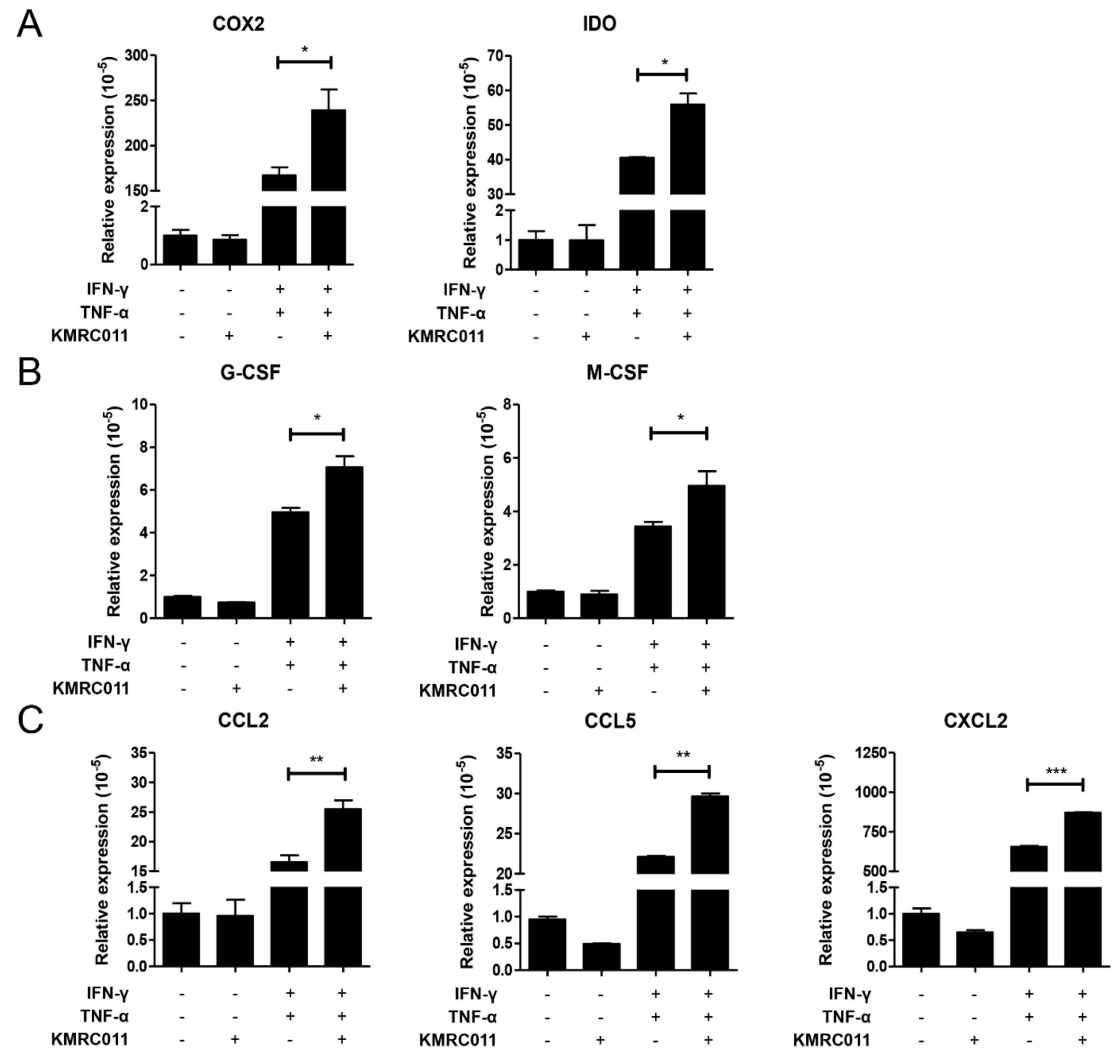
** Changes in the expression of cytokines secreted by MSCs due to increased NFκB signaling.** MSCs were cultured for 24 h with or without TLR5 agonist (100 ng/mL), IFN-γ and TNF-α at 5 ng/mL to create a low-grade inflammation. Then, qRT-PCR was used to identify immunosuppressive markers of MSCs **(A)** COX2 and IDO, **(B)** M2 macrophage activation-related markers G-CSF and M-CSF, and **(C)** M2 macrophage migration markers CCL2, CCL5, and CXCL2. The columns represent the mean values of three independent experiments and the error bars represent standard deviations (SDs). *p < 0.05, **p < 0.01, ***p < 0.001.

In our previous data, the authors observed that KMRC011-primed MSCs increased NFκB signal, and our results confirm that increased NFκB in MSCs increases immunosuppressive factors, macrophage polarization and migration-related cytokines. Figure 2A shows that the immunosuppressive effect of MSCs was increased by elevated COX2 and IDO, and Figure 2B shows that the secretion of cytokines that promote polarization to M2 macrophages, such as G-CSF and M-CSF, was also increased. Figure 2C shows the expression of cytokines involved in macrophage migration, with C-C motif ligand (CCL)2, CCL5, C-X-C motif ligand 2 (CXCL2), etc. were most increased by KMRC011-primed MSCs when a low-grade inflammation was created. Taken together, these results suggest that pretreatment of MSCs with KMRC011 in a low-grade inflammatory state enhances the immunosuppressive function of MSCs and increases their polarization and migration to M2 macrophages.

### Co-culture of KMRC011-primed MSCs with T-cell receptor-stimulated PBMCs reduces T cell proliferation

In previous experiments, we identified cytokines secreted by MSCs and compared the immunosuppressive activity of KMRC011-primed MSCs to non-primed MSCs. On day 0, we added irradiated non-amplifying MSCs and PBMCs stimulated with anti-CD3/anti-CD28 to each well of a 1:8 allogeneic mixed lymphocyte reaction. On day 4, CD3-immunostained T cells were gated to determine Kiel 67, which is used as a marker for T cell proliferation. More than 70% of CD3+ cells proliferated in the absence of non-primed MSCs or KMRC011-primed MSC; however, proliferation was reduced significantly in the presence of KMRC011-primed MSCs compared to no MSCs.

### KMRC011-primed MSCs increased survival and reduced clinical scores in a GVHD model

Examination of the proportion of macrophages in the spleens of D+21 d mice revealed that the KMRC011-primed MSC group had a significantly increased proportion of M2 macrophages expressing CD206+ compared to the non-primed MSC group. To further evaluate the similarities and differences in biological function between the abovementioned *in vivo* non-primed MSC s and KMRC011-primed MSC, a general GVHD model was used. To induce the GVHD model, a mixture of 5×106 bone marrow cells (BMCs) and 5×106 spleen cells (SCs) from donor C57BL/6 mice was transplanted via the tail vein of recipient BALB/c mice after 800 cGy total body irradiation (TBI) (Figure 4A). The GVHD mice were then randomized into three experimental groups: saline-infused control (Allo), 1×106 MSCs, and 1×106 KMRC011-primed MSC. The results showed that mice systemically infused with MSCs had reduced mortality and prolonged survival compared to the control group (Allo), with KMRC011-primed MSCs showing better therapeutic effects than non-primed MSCs (Figure 4B). Recipient mice transplanted with allogeneic BMCs and SCs had lower survival and higher GVHD scores within 27 d (Figure 4B-4C). The KMRC011-primed MSC group showed greater protection against GVHD mice, with the lowest clinical scores and relief of GVHD symptoms, including weight loss (Figure 4C-4D).

### GVHD responses were attenuated in the group receiving KMRC011-primed MSCs compared to the group receiving MSCs

Histopathological evidence of GVHD in key GVHD target organs, including skin, liver, small intestine, and large intestine, identified at day 21 post-transplant (Figure 5), clearly showed that the KMRC011-primed MSC group had attenuated graft-versus-host disease compared to the non-primed MSC group. Liver samples from the disease-induced group showed inflammatory infiltration; however, the degree of infiltration was reduced in the non-primed MSC group, with the least infiltration in the KMRC011-primed MSC group. The small intestinal mucosal crypt of the disease-induced group was disrupted severely, showing proliferating and hyperchromatic nuclei, and numerous cells exhibited apoptotic characteristics. The villus length of the small intestine was longer in the KMRC011-primed MSC group than in the MSC group. The expression levels of T cells were evaluated by immunohistochemistry in the small intestine of mice at day 21 post-BMT. The results showed that the levels of Th1 expressing IFN-γ were reduced significantly in the small intestine of KMRC011-primed MSCs compared to non-primed MSCs. In addition, the levels of Treg expressing FoxP3 were increased significantly by KMRC011-primed MSCs compared to non-primed MSCs.

### KMRC011-primed MSCs increased the M2 ratio and induced migration of macrophages in the spleen of a GVHD model

We also isolated the spleens of the mice on days 21 and 28 to determine the proportions of macrophages. On day 21, the non-primed MSC group exhibited a higher proportion of M1 macrophages, while the KMRC011-primed MSC group showed an increased proportion of M2 macrophages (Figure 6A-6D). The M1 to M2 ratio of macrophages was the highest in the KMRC011-primed MSCs group (Figure 6B). By day 28 post-treatment, a significant decrease in the proportion of M1 macrophages (Figure 6E-6G) and a significant increase in the proportion of M2 macrophages were observed in the KMRC011-primed MSC group compared to the non-primed MSC group (6E-6F, 6H). The results demonstrate that KMRC011-primed MSCs can alleviate GVHD by increasing the proportion of M2 macrophages. Furthermore, as the treatment progressed, there was a further decrease in the M1/M2 ratio.

## Discussion

The ability of MSCs to reduce variability and exert immunomodulatory effects in low grade inflammation shows great promise for MSC-based therapies in the treatment of steroid-refractory GVHD. Although several clinical trials have demonstrated the efficacy of MSCs in steroid-refractory GVHD, 29, 30 other studies have reported conflicting results. 4 Variability in MSC populations may be one reason for the inconsistent results, 31 which highlights the importance of minimizing MSC variability and maintaining and enhancing their immunomodulatory activity in low grade inflammation.

The immunomodulatory properties of MSCs have raised safety concerns with regard to an increased risk of primary viral infection and viral reactivation, which is a major cause of mortality after allogeneic HSCT. 32, 33 However, the interaction between MSCs and viruses also has beneficial effects, including promoting the proliferation and function of antiviral-specific effector cells, making MSCs a valuable tool for studying viral pathogenesis, and protecting the host against viral challenges through their antimicrobial activity. 34 A more individualized and detailed understanding of the level of immunosuppression in each patient infused with MSCs would facilitate more accurate stratification of infection risk. Such a personalized approach is critical to optimizing infection control and prevention in HSCT recipients.

In this study, we sought to develop an MSC-based therapy by pretreating MSCs with KMRC011, a TLR5 agonist. Various attempts have been made to eliminate the variability of MSCs and use them as therapeutic agents, even in low grade inflammation. These include gene transfer, 35 co-transplantation with other highly immunomodulatory cells, 36, 37 and stimulation with substances such as cytokines. 15 In this study, we found that priming with a TLR5 agonist allowed MSCs to act as a therapeutic agent even in low grade inflammation, which is the predominant environment in a clinic, and induced polarization of M2 macrophages, which was not found in previous studies, alleviating GVHD.

Furthermore, TLR5 is predominantly expressed on innate immune cells, such as dendritic cells and macrophages. 38 However, it is rarely expressed in lymphocytes 39, suggesting that it does not affect cells directly involved in immune regulation. We investigated the mechanism of action of MSCs in secreting cytokines and altering the inflammatory environment.

Priming MSCs with KMRC011 increases various cytokines that are involved in immune regulation or polarize or recruit M2 macrophages after an increase in NFkB signal, which can be time point dependent when MSCs are stimulated with KMRC011. In the present study, cells were harvested at 24 h, when cytokine secretion is at its peak, to determine the expression of NFkB and cytokines. In a follow-up experiment, it will be interesting to check the NFkB signal and cytokine expression of MSCs after priming with KMRC011 at various time points.

**Figure 3:**
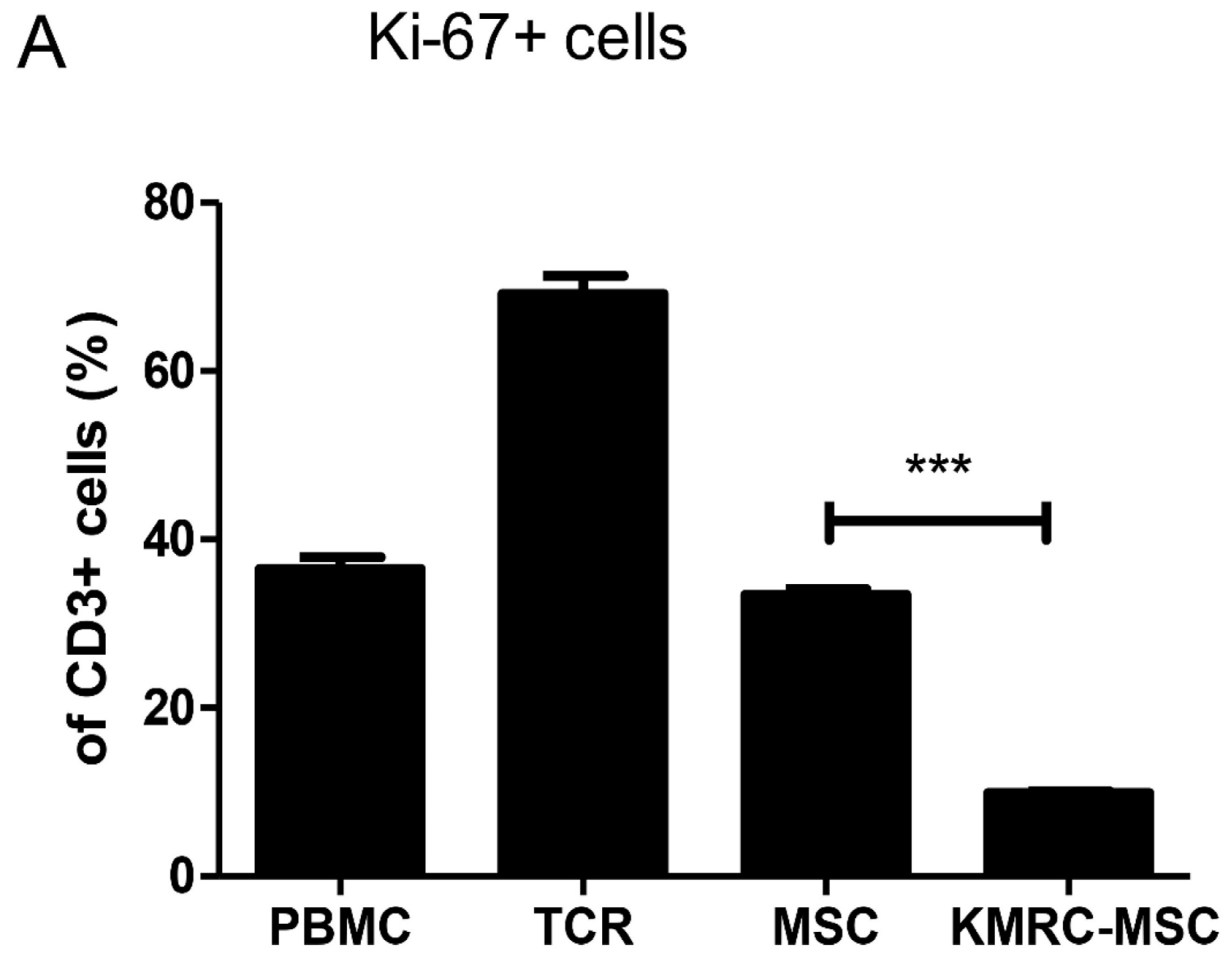
** PBMC co-cultured with MSC or TLR5 preconditioned MSC using 1:8 ratio of MSCs/PBMCs.** MSCs and PBMCs irradiated with 2,000 rad were co-cultured at a 1:8 ratio in 96-well plates treated with anti-CD3/anti-CD28 for T-cell proliferation. **(A)** The proportion of cells expressing CD3+, Ki-67+ was assessed by flow cytometry on day 4. Representative FACS data and mean expression for three different matched lots of MSCs and MSCs stimulated with TLR5 agonist are shown. The columns represent the mean values of three independent experiments and the error bars represent standard deviations (SDs). *p < 0.05, **p < 0.01, ***p < 0.001.

**Figure 4:**
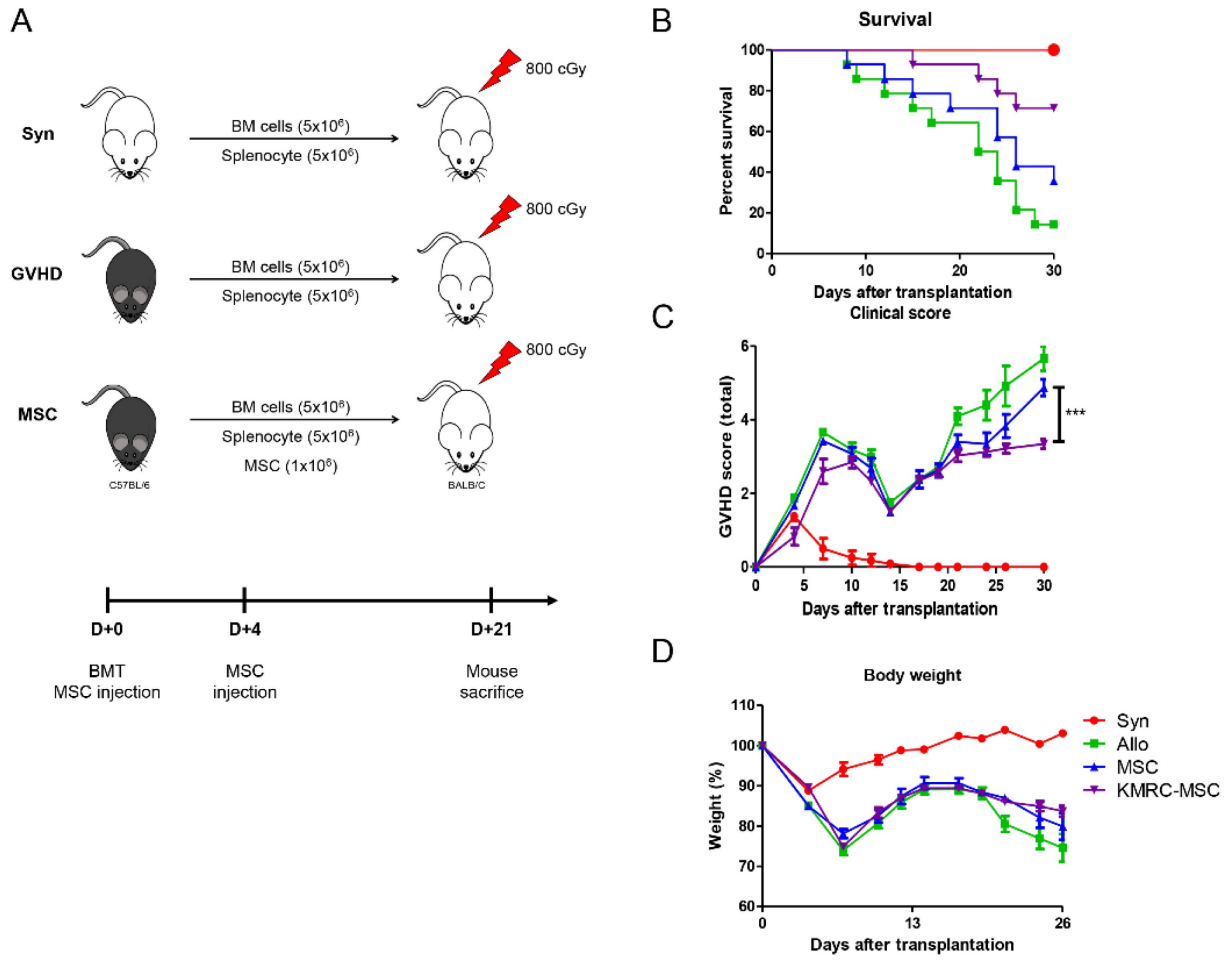
** KMRC-MSC improves severity of GVHD.** Mesenchymal stem cells treated with a TLR5 agonist showed superiority in therapeutic effect on GVHD mice *in vivo*. The GVHD mouse model was induced in recipient BALB/c mice treated with 800 cGy total body irradiation (TBI). Bone marrow cells (BMCs) and spleen cells (SCs) were harvested from C57BL/6 donor mice, and then BMCs and SCs were mixed 1:1 at 5×10^6^ each and administered by tail vein injection under sterile conditions. aGVHD mice were randomly divided into control (1×PBS injection) and experimental groups (GVHD+MSCs, GVHD+KMRC-MSCs). MSC or KMRC-MSC administration was performed twice on days 0 and 4. **(A)** Mock-up of GVHD induction and treatment groups. **(B)** Evaluation of survival rate of mice between groups **(C)** Evaluation of GVHD disease score of control and experimental groups (GVHD+MSC, GVHD+KMRC-MSC) **(D)** Evaluation of body weight of mice. The columns represent the mean values of four independent experiments and the error bars represent standard deviations (SDs). *p < 0.05, **p < 0.01, ***p < 0.001.

**Figure 5:**
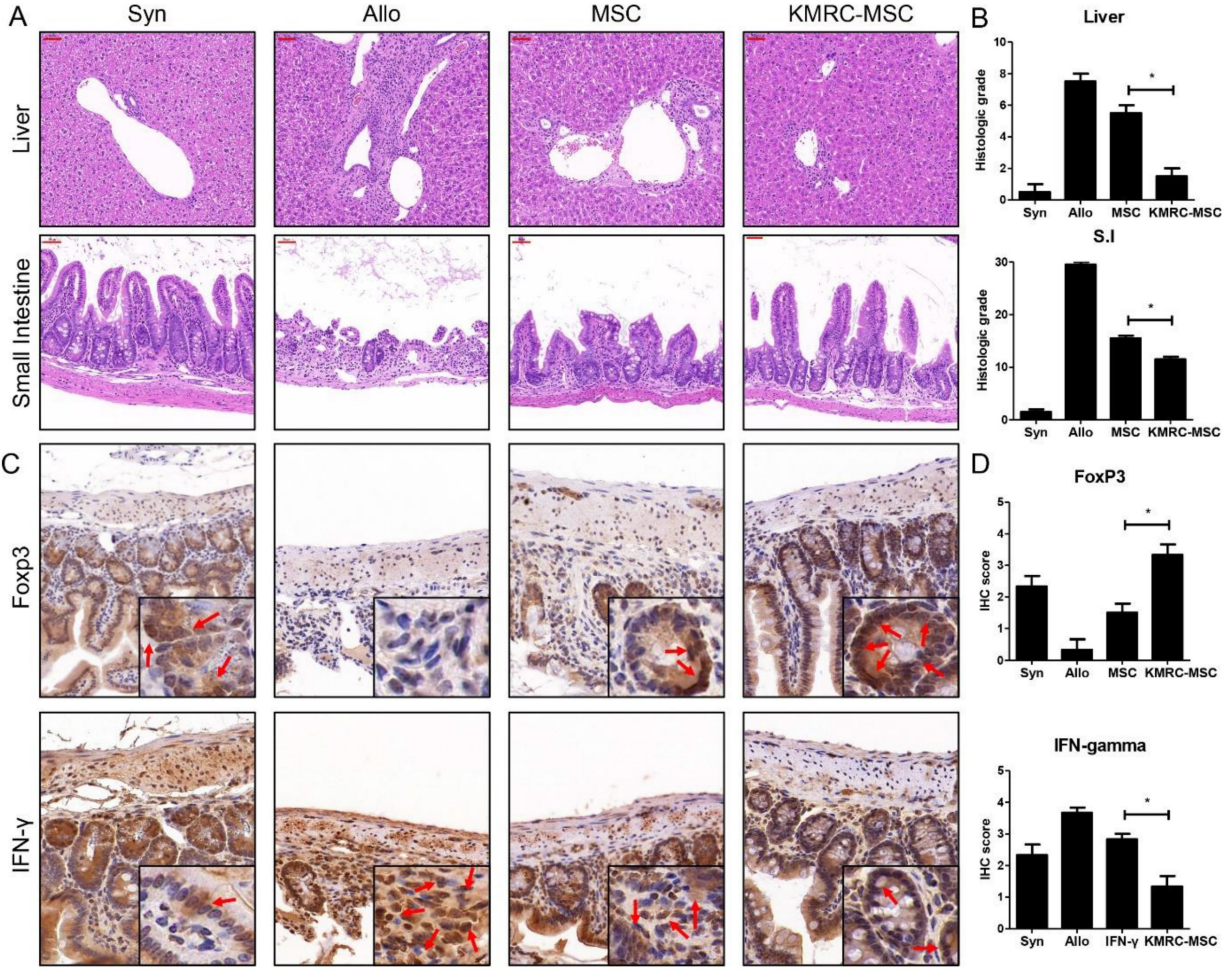
** KMRC011-primed MSCs attenuate the GVHD response. (A)** Histological tissue sections of the liver and small intestine were obtained from experimental mice on day 21 (original magnification, ×100 for liver and small intestine). **(B)** Liver and small intestine slides from mice at day 21 post-transplant were stained with hematoxylin and eosin, and GVHD severity was expressed as a quantitative graft-versus-host disease histopathology score according to standard criteria. The GVHD induction score was reduced significantly in the group receiving KMRC-MSCs at day 21 post-transplant. **(C-D)** IHC staining at 20× magnification detected IFN-γ and FoxP3 in the small intestine on day D+21 after GVHD induction. **(C)** The levels of Th1 expressing IFN-γ were decreased significantly in the small intestine in the KMRC011-primed MSC treatment compared to the non-primed MSC treatment group. In addition, the levels of Treg expressing FoxP3 were increased significantly by the KMRC011-primed MSC treatment group compared to the non-primed MSC treatment group. **(D)** The score graph was expressed as a quantitative IHC grade. Data are presented as mean ± standard error. *P<0.05, compared to the GVHD group (n = 14).

The results showed that treatment of MSCs with TLR5 agonists enhanced their immunosuppressive function *in vitro*: the immunosuppressive markers IDO, 40, 41 and COX2 42 were increased in MSCs pretreated with TLR5 agonists compared to non-primed MSCs, and they inhibited T cell proliferation most efficiently (Figure 3). MSCs can also regulate immunity by secreting cytokines and affecting immune cells *in vivo*. 43 In the present study, the strongest immunosuppressive effect was observed when MSCs were pretreated with TLR5 agonists, which could be attributed to an increase in the immunosuppressive markers IDO and COX2, as well as increases in M-CSF and G-CSF, one of the cytokines that induces polarization into M2 macrophages.

*In vivo*, the efficacy of MSCs in the treatment of aGVHD was enhanced in the group receiving MSCs pretreated with TLR5 agonists, with lower aGVHD clinical scores and improved survival. aGVHD is a systemic disease that involves numerous organs, including the small intestine, liver and skin. 44 MSCs can migrate to damaged tissue, attenuating the severity of inflammation and promoting tissue repair. 45 After intravenous injection, MSCs pretreated with TLR5 agonists can migrate to the injured target organ and induce the recruitment of M2 macrophages to the lesion site. The present study demonstrated that MSCs stimulated with TLR5 agonists are excellent mediators for delaying the onset and reducing the severity of aGVHD, suggesting a novel strategy for the treatment of aGVHD.

**Figure 6:**
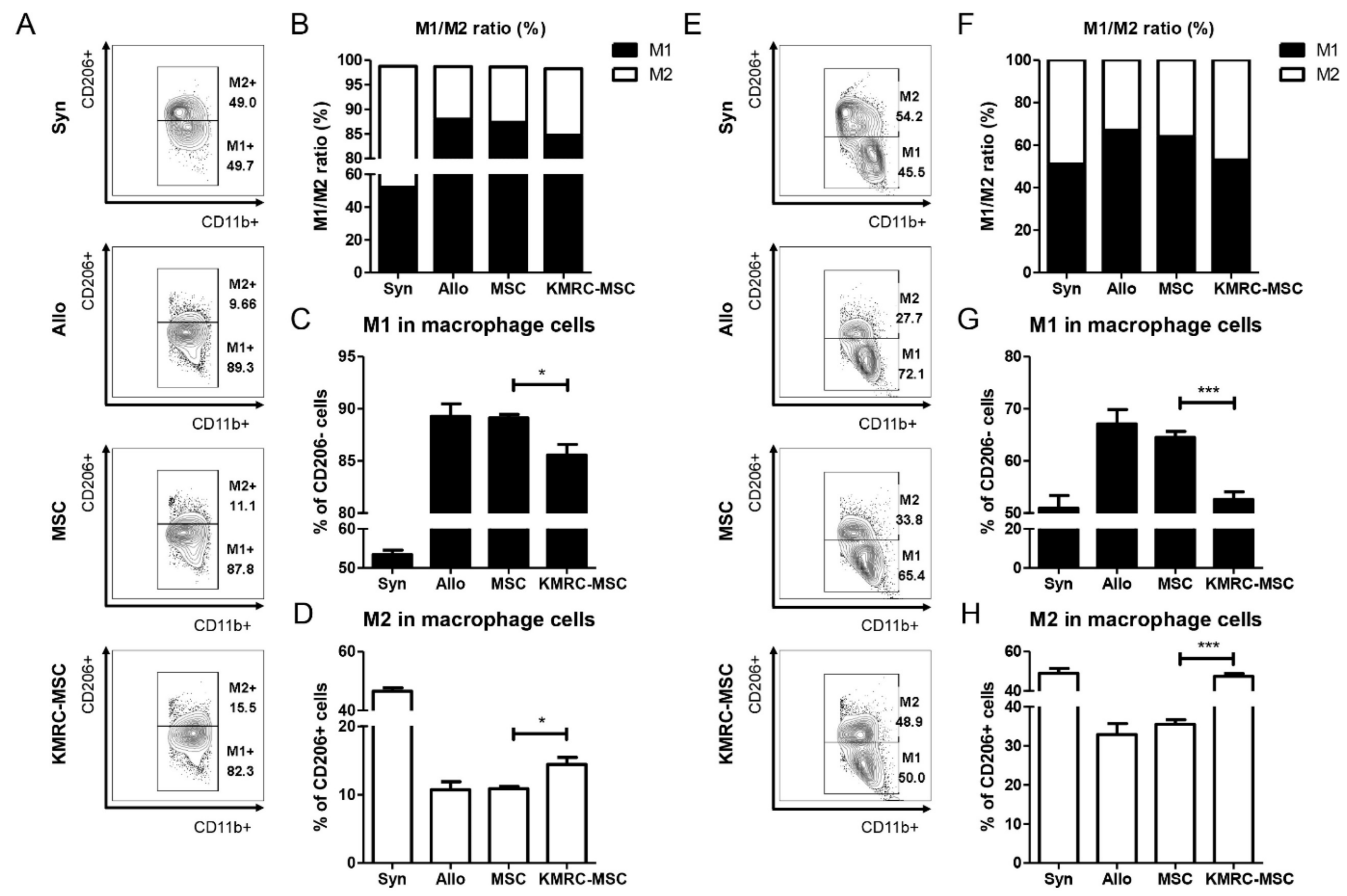
** Macrophage ratio in the spleen of mice sacrificed on day D+21 and D+28 show an increase in M2 ratio when treated with KMRC011 compared to MSCs. (A)** FACS data from isolated spleens of D+21 mice to measure M1/M2 macrophage ratio and identify polarization-induced changes in cytokine expression **(B)** Visualization of FACS data to measure M1/M2 ratio at D+21. **(C)** Graph showing the percentage of M1 macrophages at D+21. **(D)** Graph showing percentage of M2 macrophages at D+21. **(E)** FACS data from isolated spleens of D+28 mice to measure the M1/M2 macrophage ratio and identify polarization-induced changes in cytokine expression **(F)** Visualization of FACS data to measure M1/M2 ratio of D+28. **(G)** Graph showing proportion of M1 macrophages at D+28 **(H)** Graph showing proportion of M2 macrophages at D+28. Columns represent mean values of four independent experiments and error bars represent standard deviation (SD). *p < 0.05, **p < 0.01, ***p < 0.001.

Macrophages are broadly categorized into pro-inflammatory macrophages (M1) and anti-inflammatory macrophages (M2). Mesenchymal stem cells are known to convert macrophages to the M2 subset. 46 In the case of cGVHD, CD163 expressed by M2 macrophages is a macrophage scavenger receptor and is elevated in oxidative conditions. Such results suggest that monocyte or macrophage activation or increased oxidative stress may contribute to cGVHD pathogenesis. 47 Although the role and mechanism of macrophages in aGVHD is not yet clearly understood, previous studies have shown that treatment of BM obtained from healthy donors with G-CSF, a cytokine that polarizes them to M2 macrophages, followed by transplantation, reduces the M1/M2 ratio and may contribute to prevention of the development of grade 2-4 aGVHD in patients after allo-HSCT. 48 In the present study, we found an increased proportion of M1 macrophages in the aGVHD model, a slight decrease in M1 macrophages in the non-primed MSCs group, and the greatest decrease in M1 macrophages in the KMRC011-primed MSCs group. The M2 macrophage polarization-inducing cytokines IL-4, IL-10, and M-CSF secreted by mesenchymal stem cells polarize Mϕ into M2 macrophages, and these macrophages have therapeutic potential for GVHD. 49 In the present study, MSC priming with TLR5 agonists as a cell therapy resulted in the upregulation of cytokines that increased the differentiation of M2 macrophages (Figure 6). Overall, the results may partially explain why infusion of MSCs pretreated with TLR5 agonists increases M2 macrophages in the murine aGVHD model. However, the exact underlying mechanisms of M2 macrophage polarization and function are very elaborate and unclear in the present study and require further elucidation.

MSCs have been clinically applied to treat various autoimmune and inflammatory diseases. MSCs themselves secrete various cytokines; however, their interaction with other immune cells can be used as a treatment for various diseases. 50 Unlike MSCs that operate in a high inflammatory environment, KMRC011-primed MSCs can function as a therapeutic agent in a low inflammatory environment, as well as polarize and migrate M2 macrophages to achieve inflammation and disease alleviation effects in target organs.

In conclusion, the findings of the present study suggest that TLR5 agonists can affect the activity of NFκB signaling in mesenchymal stem cells, increase the secretion of anti-inflammatory cytokines, and induce their polarization into M2 macrophages, which may induce long-term survival of GVHD models and improve therapeutic efficacy.

## Figures and Tables

**Table 1:** Real-time PCR primer sequences.

Gene Symbol	Full Name	Forward Primer 5'→3'	Reverse Primer 5'→3'	Amplicon Size (bp)	Annealing (°C)
β-actin	Mus musculus actin, beta	CATTGCTGACAGGATGCAGAAGG	TGCTGGAAGGTGGACAGTGAGG	138	56-60
COX-2	Mus musculus prostaglandin-endoperoxide synthase 2 (Ptgs2)	GCGACATACTCAAGCAGGAGCA	AGTGGTAACCGCTCAGGTGTTG	132	60
NFκB	nuclear factor of kappa light polypeptide gene enhancer in B cells 1	ATCAGACACCTCTGCACTTG	GTCCTTCTTTGGCAGCTAGG	135	56
IDO	Ido1 indoleamine 2,3-dioxygenase 1	CCTGCAATCAAAGCAATCCC	GTGTCTGGGTCCACAAAGTC	149	56
CCL2	Mus musculus chemokine (C C motif) ligand 2	CCTGCTGCTACTCATTCACC	CTGGACCCATTCCTTCTTGG	155	56
CCL5	Mus musculus chemokine (C C motif) ligand 5	AGGAGTATTTCTACACCAGCAG	CTTCTTCTCTGGGTTGGCAC	95	56
M-CSF	Mus musculus colony stimulating factor 1 (macrophage) (Csf1)	CAATGCTAACGCCACCGAG	ATGGAAAGTTCGGACACAGG	106	57
G-CSF	Mus musculus colony stimulating factor 3 (granulocyte) (Csf3)	AACTTTGCCACCACCATCTG	GAAGTGAAGGCTGGCATGG	92	56
CXCL2	Mus musculus chemokine (C X C motif) ligand 2	CCAGAGCTTGAGTGTGACG	GTTAGCCTTGCCTTTGTTCAG	151	56
